# Evaluation du niveau d'activité physique dans un service Marocain d'hémodialyse chronique

**DOI:** 10.11604/pamj.2013.15.79.1830

**Published:** 2013-06-29

**Authors:** Ilham Karimi, Nawal Benabdellah, Yassamine Bentata, Hicham Yacoubi, Intissar Haddiya

**Affiliations:** 1 Service de Néphrologie-Dialyse, Hôpital Al Farabi, Université Mohamed Premier, Faculté de médecine et de pharmacie, Oujda, Maroc; 2 Service d'Orthopédie, Hôpital Al Farabi, Université Mohamed Premier, Faculté de médecine et de pharmacie, Oujda, Maroc

**Keywords:** Activité physique, Cardiopathie, Douleur, Faiblesse musculaire Hémodialyse, Hyperparathyroïdie secondaire, Inflammation, Insuffisance rénale chronique, Qualité de vie, sujet âgé, physical Activity, cardiopathy, pain, muscle weakness, secondary hyperparathyroidism, inflammation, Chronic renal failure, Quality of life, elderly

## Abstract

**Introduction:**

L'activité physique est souvent réduite chez le patient hémodialysé chronique. Plusieurs études ont montré l'intérêt du maintien ou de la reprise d'une activité physique sur l’état de santé des patients. Le but de notre travail est d'analyser le niveau d'activité physique chez nos patients hémodialysé chronique.

**Méthodes:**

Nous avons réalisé une étude transversale au centre d'hémodialyse de l'hôpital Al Farabi d'Oujda. Nous avons évalué l'activité physique de nos patients à l'aide du Questionnaire de Baecke traduit en dialectal Marocain et adapté au contexte socioculturel Marocain. Nous avons relevé les paramètres démographiques et clinico-biologiques ainsi que le niveau d'activité physique. L'analyse statistique est réalisée par le logiciel SPSS 11.5.

**Résultats:**

Notre étude a inclus 83 patients. L’âge moyen était de 47,3±13,2 ans, avec une prédominance masculine. 15,6% déclarent n'avoir aucune activité physique. 68% des patients rapportent des difficultés à effectuer des efforts physiques importants (courir, soulever un objet lourd). 16,4% des patients déclarent avoir une activité physique et sportive régulière (marche, foot-ball etc..). La diminution de l'activité physique est corrélée de façon significative à l’âge avancée, l'indice de masse corporelle bas, l'ancienneté en hémodialyse et les affections cardio-vasculaires.

**Conclusion:**

Un programme d'activité physique adapté à notre population d'hémodialysés et élaboré en concertation pluridisciplinaire (cardiologues, orthopédistes, rhumatologues, kinésithérapeutes) paraît intéressant dans le cadre d'une prise en charge globale du patient hémodialysé chronique.

## Introduction

L'insuffisance rénale chronique terminale (IRCT) traitée par hémodialyse (HD) connaît une augmentation considérable de part le monde. Au Maroc, le nombre de personnes atteintes d'IRCT et prises en charge dans les 157 centres d'hémodialyse publics et privés du royaume atteint 7850 [1]. En effet, l'HD a complètement transformé l’évolution de l'insuffisance rénale chronique (IRC) permettant d'améliorer le confort des patients et leur espérance de vie. Toutefois, en tant que traitement palliatif, l'HD est vécue comme une expérience pénible et contraignante du fait de la lourdeur des complications qui lui sont inhérentes et de la morbi-mortalité qui en résulte [2].

La faiblesse musculaire et l'asthénie excessive sont parmi les complications préoccupantes mais peu étudiées chez les patients atteints d'IRC, en particulier ceux qui sont dialysés. Elles conduisent à une activité physique réduite, qui peut avoir des conséquences graves. Plusieurs études ont pu mettre en évidence une relation inverse entre le niveau d'activité physique et la pathologie obstructive coronarienne [3]. Elles ont ainsi démontré l'intérêt du maintien ou de la reprise d'une activité physique sur l’état de santé des patients dialysés ainsi que leur qualité de vie. De plus, la sédentarité, qui est reconnue comme un facteur de risque classique de maladie cardiovasculaire, est associée à un risque de surmortalité chez la population dialysée [4]. Il existe de nombreux outils de mesure de l'activité physique et l'aptitude physique. Le questionnaire de Baecke tient sa pertinence du fait qu'il permet de construire trois indices représentatifs: indice d'activité de travail, indice d'activité sportive, et indice d'activité de loisir [5]. L'objectif de notre étude était d'analyser le niveau d'activité physique chez nos patients HDC et de dégager les facteurs de risque d'une activité physique réduite.

## Méthodes

Nous avons réalisé une étude transversale, en Novembre 2011, incluant 83 patients hémodialysés chroniques de l'Hôpital Al Farabi d'Oujda, dans la région de l'Oriental Marocain.

**Choix de l'outil de mesure de l'activité physique** Nous avons évalué l'activité physique de nos patients à l'aide du Questionnaire de Baecke, c'est un questionnaire auto-administrable. A partir de 16 questions, trois indices sur une échelle de 1 à 5, représentatifs de l'activité physique habituelle du sujet, sont obtenus: un indice d'activité de travail (IAT), un indice d'activité sportive (IAS), un indice d'activité de loisir (IAL) [5–7].

La mise au point du questionnaire cible a comporté plusieurs étapes afin de l'adapter à notre contexte culturel Marocain. La méthodologie d'adaptation que nous avons suivie peut être résumée de la façon suivante:

- Traductions indépendantes (du Français vers le Dialectal Marocain), préalablement à la traduction de synthèse qui est réalisée par un groupe de professionnels qui représentent des compétences de différentes disciplines (Néphrologues, psychosociologues, Enseignants de littérature française...etc).
- Modification du questionnaire en fonction de l’équivalence de la version Arabe Dialectale par rapport au questionnaire original d'une part, et en fonction des remarques et incompréhensions des patients HD ayant participé au pré-test.
- Contre-traduction vers le français.

Le questionnaire a été administré par les médecins du service au cours des séances d'hémodialyse. L'auto-administration n’était pas possible dans notre contexte vu le grand nombre de patients analphabètes. Par ailleurs nous avons analysé chez nos patients les paramètres suivants:

**Démographiques:** à savoir l’âge, le sexe, le niveau d'instruction et les catégories socioprofessionnelles (1-Agriculteur exploitant 2-artisan commerçant ou chef d′entreprise 3-cadre profession intellectuelle supérieure, 4-profession intermédiaire, 5-employé, 6-ouvrier, 7-retraité, 8-inactif autre que retraité, 9-chômeur n′ayant jamais travaillé, 10-étudiant ou scolarisé).

**Cliniques:** Antécédents médico-chirurgicaux, la néphropathie initiale, les comorbidités (hypertension artérielle (HTA) définie par une pression artérielle systolique> 140mmhg et/ou une pression artérielle diastolique > 90mmhg; Diabète; Maladie cardio-vasculaire; néoplasie; Maladie de système évolutive; Indice de masse corporelle (IMC) défini selon l'OMS: Le surpoids correspond à un IMC égal ou supérieur à 25; de même que l'obésité correspond à un IMC égal ou supérieur à 30), les différents traitements en cours (traitement antihypertenseur, agents stimulants de l’érythropoïèse, supplémentation par le fer, calcium, chélateur de phosphore, analogues de la vitamine D).

**Biologiques:** Hémoglobine (L'anémie défini par une baisse de l'hémoglobine (Hb) en dessous de 11 g/dl). Bilan phospho-calcique (calcémie, phosphorémie, PTH intacte, 25OH vitamine D), bilan lipidique (cholestérol total, triglycérides), bilan inflammatoire (VS, CRP), réserves alcalines.

**Dialytiques:** ancienneté en HDC, nombre de séance / semaine, la qualité de dialyse Kt/V(sp). Nos patients sont tous dialysé par des générateurs 4008 (Fresenius). Le bain de dialyse utilisé est un bain standard, le tampon utilisé est le bicarbonate, avec une teneur en calcium à 1,75mmol/l, potassium à 2meq/l, et Glucose à 0, 3g/l. L'analyse statistique a été réalisée par le logiciel SPSS 11.5. Les variables quantitatives sont exprimées en moyenne±écart-type et les variables qualitatives en pourcentage. Une valeur p < 0,05 est considérée signi'cative.

## Résultats

### Données démographiques, clinico-biologiques et dialytiques de nos patients HDC

**Figure 1:**
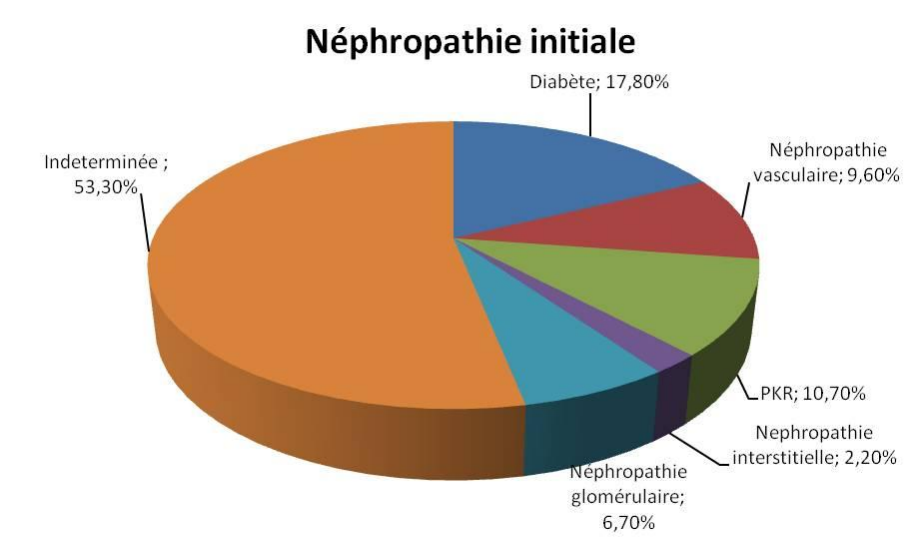
Néphropathies causales chez nos patients hémodialysés chroniques

Notre étude a inclus les 83 patients HDC dans notre centre. L’âge moyen de nos patient était de 47,3±13,2 ans, 33,7% des patients sont âgés plus de 65 ans, une prédominance masculine a été noté avec un sexe ratio de 45H/38F. Sur le plan socioprofessionnel, 67(80%) patients appartiennent à la catégorie 9, 10 (12%) patient à la catégorie 8, alors que deux patients sont de la catégorie 10. Concernant le niveau d'instruction 74 patients sont analphabètes, tandis que 9 patients sont scolarisés, six avaient le niveau du primaire, 2 autres celui du secondaire, et un seul patient poursuit des études universitaires. La néphropathie causale était diabétique dans 17,8% des cas, vasculaire dans 9,6% des cas, et glomérulaire dans 6,7% des cas. Elle était indéterminée (NI) dans 53,3% (Figure 1). 20% de nos patients avaient une cardiopathie. L'IMC était inférieur à 18% chez 19% des patients, Sur le plan biologique 80% des patients étaient anémiques, avec une hémoglobine moyenne à 9,8 ± 1,8. 56,7% des patients avaient une hyperparathyroidie secondaire avec une PTH 1-84 moyenne de 508 ± 380 pg/ml, les paramètres biologiques des patients sont résumés dans le Tableau 1. Sur le plan thérapeutique, 50% des patients recevait un traitement antihypertenseur, 64 patients étaient sous ASE. L'alfacalcidiol était prescrite chez 40,9% des patients, la vitamine D native était prescrite chez 3% des patients. Concernant les données dialytiques de nos patients, la durée moyenne de dialyse était de 102,4±41,9 mois. 53 de nos patients avaient un rythme de 2 séances par semaine, tandis que les 30 restants étaient dialysés à raison de 3 fois par semaine.

**Table 1:** Paramètres biologiques

Paramètres biologiques sanguins	Moyenne ± Ecart-type (ET)
Hémoglobine (g/dl)	9,8± 1,8
PTH1-84 (pg/l)	508± 380
Calcémie (mg/l)	85,06±12,02
Phosphorémie (mgl)	61,16 ±10,44
25 OH Vitamine D	23,9 ± 6,2
Phosphatases alcalines (UI/l)	87,8 ±18
Cholestérol	2,01 ± 0,9
Triglycérides	1,90 ± 0,74

### Activité physique de nos patients HDC (Figure 2)

Afin d’évaluer l'activité physique de nos patients HDC, nous avons opté pour le questionnaire de Baecke (Figure 3) que nous avons adapté à notre contexte socio-culturel Marocain. Ainsi, ce questionnaire a été administré par le personnel médical aux patients consentants au cours des séances d'HD. Sur l'ensemble de nos patients HDC, 13 (15,6%) déclarent n'avoir aucune activité physique. Il s'agit essentiellement des patients assistés, dans leurs activités quotidiennes, par une tierce personne. 68% des cas rapportent des difficultés à effectuer des efforts physiques importants (courir, soulever un objet lourd). Seuls 16,4% des patients déclarent avoir une activité physique et/ou sportive régulière (marche, football).

**Figure 2:**
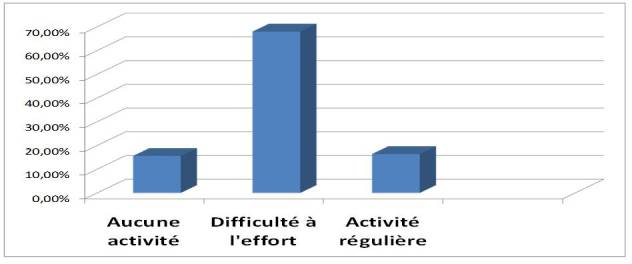
Activité physique de nos patients hémodialysés chroniques

**Figure 3:**
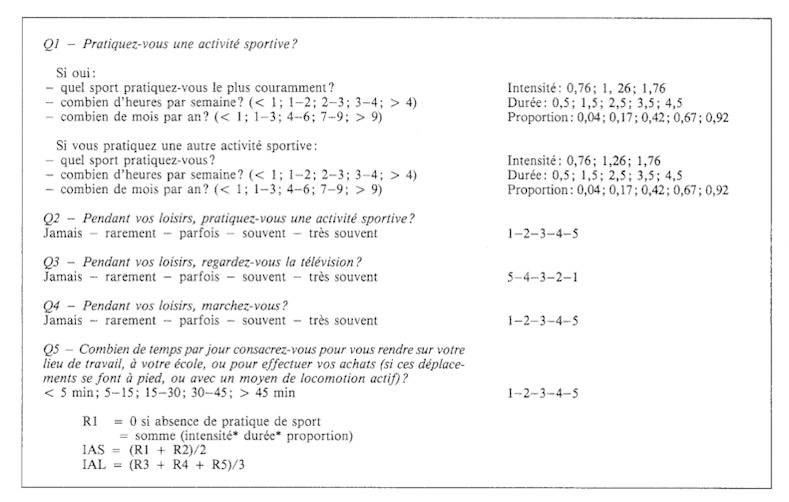
Questionnaire de Baecke

Le calcul des trois indices d'activité physique dans le cadre du Questionnaire de Baecke a permis de dégager les renseignements suivants: chez les patients qui pratiquent une activité sportive, l'indice médian d'activité sportive (IAS) est de 2,3. L'intensité médiane de cette activité sportive est de 1,26, sa durée médiane est de 1,5 heure par semaine et sa proportion médiane est de 0,67 (ce qui correspond à 7-9 mois par an). Par ailleurs, chez les patients qui exercent une activité professionnelle, l'indice médian d'activité de travail (IAT) est de 1,87. De plus, l'indice médian d'activité de loisir (IAL) est de 2,6. L'ensemble de ces 3 indices correspond à une activité physique limitée, et ce dans les différents aspects du quotidien de nos patients HDC.

### Facteurs de risque d'une activité physique réduite

Nous avons étudié la relation entre la baisse de l'activité physique et différents paramètres démographiques, cliniques et biologiques (Tableau 2). Il en ressort qu'en analyse univariée, la diminution de l'activité physique est corrélée de façon significative à l’âge avancée (p= 0,02), l'IMC bas (p=0,0001), l'ancienneté en hémodialyse ( p=0,003)et les affections cardio-vasculaires, en revanche aucune corrélation significative n'a été trouvée entre le sexe, le diabète et la diminution de l'activité physique.

**Table 2:** Facteurs de risque d'activité physique réduite chez nos patients hémodialysés chroniques

Paramètres	Activité réduite (83,6%)	Activité régulière (16,4%)	P
Age avancé (%)	34%	16.6%	0.02
Sexe ratio H/F	1.2	0.9	NS
Index de masse corporel moyen (Kg/m^2^)	20.1±3.4	23.09±2.13	0.0001
Ancienneté moyenne en HDC (mois)	126.2±34.2	97.1±21.5	0.003
Comorbidités cardio-vasculaires (%)	54%	15%	0.01
Anémie (%)	87%	25%	0.001
Hyperparathyroidie secondaire (%)	46%	15%	NS
Diabète (%)	22%	18%	NS

## Discussion

Le nombre des patients traités par HDC ne cesse de s'accroître et de vieillir, surtout avec l'amélioration des techniques d'HD et la meilleure accessibilité au traitement. Elle permet de prolonger la survie au prix de limitations fonctionnelles et d'un handicap assez importants, liés à l'insuffisance rénale, aux séances d'hémodialyse, aux comorbidités associées et à l'atteinte de l'appareil locomoteur [8–10].

Dans notre étude, seuls 16,4% des patients déclarent avoir une activité physique et/ou sportive régulière, 68% des patients rapportent des difficultés à effectuer des efforts physiques importants, alors que 13 patients (15,6%) déclarent n'avoir aucune activité physique.

La réduction de l'activité physique est un élément bien établi dans l'IRC, notamment chez le dialysé, celle-ci peur être expliquée par plusieurs facteurs:

**L'inactivité:** elle est favorisée par la maladie elle-même (état de malaise lié à la maladie rénale chronique), les effets secondaires de la dialyse et les maladies associées.

**L'atteinte musculaire:** L'atrophie musculaire est très fréquente chez les patients ayant une insuffisance rénale terminale. Elle peut être la conséquence d'une acidose, d'une corticothérapie, d'un épisode de stress oxydatif ou d'un déséquilibre métabolique en rapport avec l'hémodialyse. Un autre facteur est l'absence d'utilisation des muscles en raison d'un ralentissement des activités quotidiennes ou la non participation aux programmes d'entraînement musculaire par crainte de troubles liés à la maladie rénale (crampes, myoclonie, fatigue) ou à des périodes d'inactivité prolongées pendant l'hémodialyse. Il en résulte une réduction de la capacité fonctionnelle et une détérioration de la qualité de vie [11].

**Les douleurs:** Le retentissement de la douleur chronique sur la qualité de vie des patients est actuellement prouvé par plusieurs auteurs [2]. La douleur est responsable d'une gêne de l'activité quotidienne dans 67,7% des cas [12]. Ces douleurs peuvent s'expliquer par l'amylose b2 microglobuline. La présence de dépôts amyloïdes ‘brillaires principalement dans les tissus articulaires, para-articulaires (synoviales, tendons, ligaments) et dans les os, provoque cliniquement l'apparition de syndromes douloureux articulaires et périarticulaires et de syndromes canalaires [13].

**L'ostéodystrophie rénale**, terme générique englobant l'ostéite fibreuse, l'ostéopathie adynamique, et l'ostéomalacie, est une autre cause de douleur chez les dialysés.

**L'inflammation** est aussi un facteur qui limite l'activité physique, les causes de l'inflammation en dialyse sont nombreuses: La maladie rénale initiale peut déjà être une source d'inflammation. La diminution du catabolisme rénal des cytokines pro-inflammatoires parallèle à la diminution du débit de filtration glomérulaire, pourrait accroître l'intensité des réactions inflammatoires. L’état urémique peut activer la réponse immunitaire innée. L'exposition aux germes est aussi facilitée par les accès de dialyse, et les « bio-films » des cathéters ou des circuits extracorporels. La bio-incompatibilité des matériaux et des solutions de dialyse reste un facteur important contribuant à l'inflammation chronique du dialysé [14].

La diminution ou l'absence d'activité physique peut avoir des conséquences graves: une étude réalisée chez des patients hémodialysés, a montré que ceux qui étaient sédentaires avaient un risque de décès à un an, 62% plus élevé que ceux qui étaient plus actifs [5].

L'exercice physique augmente la résistance osseuse en majorant la densité minérale osseuse (DMO) et en améliorant la qualité du tissu osseux (géométrie et microarchitec- ture), il réduit également les risques de chutes en augmentant la force musculaire et en améliorant la stabilité posturale [15, 16].

Si l'intérêt de l'exercice physique chez les patients dialysés est connu depuis une trentaine d'années (récupération musculaire; amélioration de la fonction cardiaque; diminution de la pression artérielle; réduction de «mauvais» cholestérol (LDL) et des triglycérides, et augmentation du «bon» cholestérol (HDL), il n'y a pas aujourd'hui de recommandations établies dans ce domaine, ni de programmes structurés disponibles dans les centres, en dehors de quelques rares initiatives isolées [16].

## Conclusion

L'activité physique réduite est un signal d'alarme qui révèle le plus souvent la lourdeur des complications du patient dialysé. Un programme d'activité physique adapté à notre population d'hémodialysés et élaboré en concertation pluridisciplinaire (cardiologues, orthopédistes, rhumatologues, kinésithérapeutes) paraît intéressant dans le cadre d'une prise en charge globale du patient HDC.
